# Atherogenic index of plasma as a simple marker for NAFLD risk stratification in community-dwelling older adults: a cross-sectional study with incremental predictive value assessment

**DOI:** 10.3389/fendo.2026.1907673

**Published:** 2026-08-26

**Authors:** Mengyuan Xiao, Chengwen Xiao, Jiayu Zhang, Yuchen Ji, Jiayan Zhuang, Yingqi Chen, Zhihong Yuan, Siying Chen

**Affiliations:** 1 Hypertension Center, Department of Cardiovascular Medicine Center, Affiliated Hospital of Guangdong Medical University, Zhanjiang, China; 2 The First Clinical College, Guangdong Medical University, Zhanjiang, Guangdong, China

**Keywords:** atherogenic index of plasma, cross-sectional study, dose-response relationship, elderly population, non-alcoholic fatty liver disease, risk factors

## Abstract

The atherogenic index of plasma (AIP), calculated as log_10_(triglycerides/HDL-C), reflects atherogenic dyslipidemia and is associated with insulin resistance. Its continuous dose-response relationship and incremental predictive value for non-alcoholic fatty liver disease (NAFLD) beyond traditional risk factors remain undefined in community-dwelling older adults—a population with a high metabolic comorbidity burden where low-cost, integrated risk markers are urgently needed.

**Methods:**

From 5,783 residents aged ≥65 years without significant alcohol consumption recruited in Zhanjiang, western Guangdong (2023–2024), 4,390 were enrolled after exclusions. NAFLD was diagnosed by ultrasonography. AIP was examined using multivariable logistic regression, restricted cubic splines, subgroup analyses, and trend tests with comprehensive confounder adjustment. Incremental predictive value was assessed through a multidimensional evaluation framework comprising ROC curves, calibration, decision curve analysis, net reclassification improvement (NRI), and integrated discrimination improvement (IDI).

**Results:**

NAFLD prevalence was 42.7%, rising stepwise across AIP quartiles (22.4%, 36.9%, 50.1%, 61.3%; P for trend < 0.001). Each 1-unit increment in AIP was associated with a 6.13-fold higher NAFLD risk (OR 6.13, 95% CI 4.55-8.28) after full adjustment. Restricted cubic splines revealed a strictly linear, threshold-free association (P for nonlinearity = 0.714), consistent across age, residence, hypertension, and diabetes subgroups, with a statistically significant interaction by sex (P for interaction = 0.042). Adding AIP to a baseline clinical model improved discrimination (AUC from 0.786 to 0.811), calibration, clinical net benefit, and risk reclassification (categorical NRI 0.0997, IDI 0.0487; P< 0.001).

**Conclusions:**

AIP demonstrates an independent, linear relationship with NAFLD risk in community-dwelling older adults, exhibiting no discernible safe threshold. This low-cost, routine lipid-derived marker supports a “single-indicator, dual-prevention” strategy for simultaneous NAFLD screening and cardiometabolic risk stratification in primary care, challenging the conventional practice of relying solely on discrete lipid cut-offs.

## Introduction

Non-alcoholic fatty liver disease (NAFLD) has emerged as the most prevalent chronic liver disease worldwide, characterized by excessive intrahepatic lipid deposition in the absence of significant alcohol consumption, viral hepatitis, or other specific etiologies. The disease spectrum ranges from simple steatosis to non-alcoholic steatohepatitis (NASH), hepatic fibrosis, cirrhosis, and ultimately hepatocellular carcinoma (1). With the global epidemics of obesity, type 2 diabetes mellitus (T2DM), and metabolic syndrome, NAFLD has become a major public health challenge, affecting more than 25% of the general adult population and over 40% of older adults and individuals with metabolic comorbidities (2).

NAFLD is now recognized not merely as a hepatic manifestation of metabolic syndrome but as a multisystem disease that shares common pathogenic pathways with atherosclerotic cardiovascular disease (ASCVD), including insulin resistance, chronic low-grade inflammation, atherogenic dyslipidemia, and endothelial dysfunction (3). This shared pathophysiology creates a vicious “liver–metabolism–cardiovascular” cycle, wherein NAFLD independently increases the risk of incident T2DM, ASCVD, and chronic kidney disease (4), substantially amplifying the disease burden in aging populations (5, 6).

Lipid dysregulation is a central driver in the development and progression of NAFLD (7). As the principal organ governing lipid synthesis, transport, oxidation, and metabolism, the liver is exquisitely sensitive to perturbations in lipid homeostasis. An imbalance between hepatic free fatty acid (FFA) influx, *de novo* lipogenesis, and lipid disposal via oxidation or export in very-low-density lipoprotein (VLDL) particles leads to triglyceride accumulation, oxidative stress, lipotoxicity, and activation of hepatic stellate cells, ultimately promoting the transition from steatosis to steatohepatitis and fibrosis (8). Although conventional lipid parameters—including total cholesterol (TC), triglycerides (TG), low-density lipoprotein cholesterol (LDL-C), and high-density lipoprotein cholesterol (HDL-C)—are widely used in clinical practice, each reflects only a single facet of the complex lipid milieu and fails to capture the integrated atherogenic potential of the lipoprotein profile (9).

The atherogenic index of plasma (AIP), calculated as log_10_ (TG/HDL-C), is a composite biomarker that captures the imbalance between triglyceride-rich lipoproteins and HDL-C (10). Rather than simply reflecting elevated lipid levels, AIP serves as a practical indicator of “lipid partitioning dysregulation”—a state in which excess free fatty acids are directed toward non-adipose tissues, including the liver and pancreatic islets (11). This ectopic lipid deposition is a hallmark of insulin resistance and lipotoxicity (12). AIP is strongly correlated with visceral adiposity, systemic insulin resistance, and chronic low-grade inflammation, processes that lie at the core of NAFLD pathogenesis (13).Its link to both hepatic steatosis and pancreatic β-cell dysfunction positions AIP as a potential bridge marker connecting the liver, pancreas, and adipose tissue in the broader context of metabolic disease.

A growing body of evidence has linked elevated AIP with an increased risk of hepatic steatosis. However, several important gaps remain. Most published studies have been conducted in general adult or middle-aged populations, and few have specifically investigated this relationship in community-dwelling older adults aged ≥65 years, a group with a disproportionately high burden of metabolic comorbidities and age-related alterations in lipid metabolism (14). Many previous studies adjusted for only a limited set of confounders—typically age, sex, and body mass index—without accounting for important covariates such as waist circumference, inflammatory markers including C-reactive protein, and glycemic indices such as HbA1c, raising questions about the independence of the observed association (15).

Moreover, the shape of the dose-response relationship between AIP and NAFLD has rarely been formally characterized using flexible modeling approaches; whether a linear, threshold-free association exists, particularly in the elderly, remains unknown. The incremental predictive value of AIP beyond a comprehensive panel of traditional metabolic risk factors has also not been quantified in elderly-specific populations, limiting the evidence base for its clinical adoption in this demographic (16). Furthermore, in the western Guangdong region of China, the prevalence of dyslipidemia exceeds 50% and hypertension affects more than 55% of adults aged ≥60 years, driven in part by high-salt, high-fat dietary patterns. This distinctive metabolic milieu may impart unique features to the AIP–NAFLD relationship that have not yet been explored (17).

To address these knowledge gaps, the present study aimed to systematically evaluate the independent association between AIP and NAFLD in a large, community-based sample of older adults in western Guangdong (18).We also sought to characterize the dose-response relationship between AIP and NAFLD using restricted cubic splines. Additionally, we aimed to quantify the incremental predictive value of AIP beyond traditional risk factors, employing multiple contemporary performance metrics including ROC analysis, calibration, decision curve analysis, NRI, and IDI. Finally, we assessed the consistency of the association across key clinical subgroups defined by sex, age, residence, hypertension, and diabetes status (19).

## Methods

### Study design

This community-based, cross-sectional observational study was conducted among permanent elderly residents of Zhanjiang City who completed standardized basic public health examinations between January 2023 and December 2024. The primary objectives of this study were to: ①investigate the independent association between the atherogenic index of plasma (AIP) and the risk of prevalent non-alcoholic fatty liver disease (NAFLD); ②analyze the potential dose-response relationship between AIP and NAFLD in the elderly population; and ③evaluate the predictive performance of AIP for NAFLD and its incremental value beyond traditional risk factors, thereby providing methodological support for early NAFLD screening and individualized risk assessment. To achieve these objectives, two NAFLD risk prediction models were constructed, and the predictive performance and incremental value of AIP were quantified using systematic model evaluation methods to ensure the scientific validity and clinical utility of the study findings.

### Ethics statement

This study was approved by the Ethics Committee of the Affiliated Hospital of Guangdong Medical University (Approval No.: PJKT2023-162). The research process strictly adhered to medical ethical principles. All participants provided written informed consent and voluntarily participated in this study after being fully informed of the study objectives, procedures, and potential risks.

### Study population and screening process

Data for this study were derived from a routine physical examination cohort of permanent residents at community health service centers in Zhanjiang City. These centers conducted standardized basic public health examinations for local permanent residents between 2023 and 2024, systematically collecting demographic information, anthropometric measurements, abdominal ultrasonography results, and serum biochemical data from all participants. This examination program was approved by the Ethics Committee of the Affiliated Hospital of Guangdong Medical University, and all participants provided written informed consent prior to participation.

**Figure 1:**
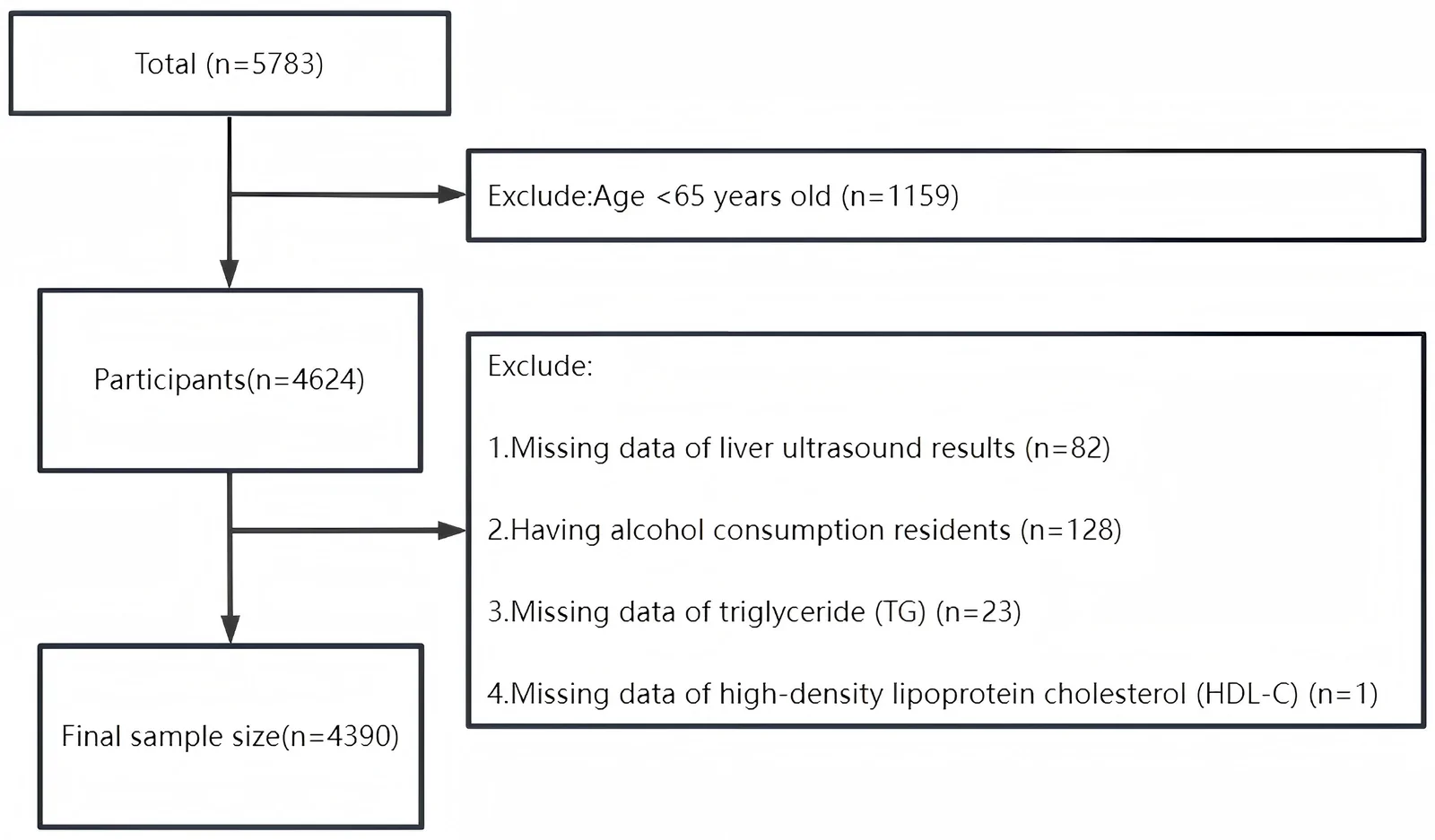
Flow chart of participant screening.

We initially enrolled a total of 5,783 permanent residents who completed routine physical examinations from the 2023–2024 cohort. Further exclusions were applied for the following reasons: aged<65 years; did not complete the abdominal ultrasound examination (n=82); average weekly ethanol intake >140 g for males or >70 g for females during the preceding year, with the excessive alcohol consumption threshold defined according to the Guideline for the Prevention and Treatment of Metabolic Dysfunction-associated Fatty Liver Disease (Version 2024) (20); missing triglyceride (TG) data (n=23); and missing high-density lipoprotein cholesterol (HDL-C) data (n=1). Ultimately, 4,390 eligible participants were included in the analysis. The detailed screening flow is depicted in **Figure 1**. The complete data of the enrolled participants were used for the subsequent construction of the two prediction models, predictive performance evaluation, and incremental value analysis of AIP, thereby providing robust sample support for the study conclusions.

The atherogenic index of plasma (AIP) is a validated composite biomarker reflecting lipid metabolism disturbances and atherogenic risk. It is calculated as follows (21, 22): $\text{AIP}=\log_{10}(\text{TG/HDL-C})$. Since serum TG and HDL-C were measured in mmol/L, they were converted to mg/dL using the factors: TG (mg/dL) = TG (mmol/L) × 88.6, HDL-C (mg/dL) = HDL-C (mmol/L) × 38.67.

### Diagnosis of NAFLD

NAFLD was diagnosed by professional sonographers using abdominal ultrasonography, with procedures strictly adhering to the American Association of Clinical Endocrinology Clinical Practice Guideline for the Diagnosis and Management of Nonalcoholic Fatty Liver Disease in Primary Care and Endocrinology Clinical Settings: Co-Sponsored by the American Association for the Study of Liver Diseases (AASLD) (23). All sonographers were blinded to the participants’ clinical and laboratory data to minimize observer bias. Diagnostic criteria included increased hepatic echogenicity, enhanced hepato-renal echo contrast, deep ultrasound beam attenuation, and blurring of intrahepatic vascular structures (24). Participants with excessive alcohol intake, viral hepatitis, autoimmune liver disease, or drug-induced liver injury were excluded (25). The accuracy of NAFLD diagnosis was ensured to lay the foundation for the validity of the prediction models.

### Covariate assessment

Trained investigators collected demographic, lifestyle, anthropometric, and laboratory data using standardized protocols, with demographic and lifestyle variables including age, sex, area of residence (urban/rural), marital status, smoking status, history of hypertension, and history of diabetes; anthropometric measurements including height, weight, body mass index (BMI), waist circumference (WC), systolic blood pressure (SBP), and diastolic blood pressure (DBP);and laboratory variables including white blood cell (WBC) count, hemoglobin (HGB) concentration, alanine aminotransferase (ALT), alkaline phosphatase (ALP), albumin (ALB), uric acid (UA), fasting blood glucose (FBG), low-density lipoprotein cholesterol (LDL-C), glycated hemoglobin (HbA1c), total cholesterol (TC), aspartate aminotransferase (AST), and C-reactive protein (CRP).

### Laboratory testing

Fasting venous blood samples were collected from all participants after an overnight fast of 8–12 hours. Samples were centrifuged at 3,000 rpm for 10 minutes to separate serum, and serum aliquots were stored at −80 °C in accordance with expert consensus recommendations for frozen erythrocyte quality assessment indicators, avoiding repeated freeze-thaw cycles (26). Serum levels of TG, HDL-C, TC, and LDL-C were measured by immunoturbidimetry (27), with all detection procedures strictly adhering to national clinical laboratory quality control standards.

### Statistical analysis

Data processing and statistical analysis followed a rigorous analytical strategy. For baseline characteristics, continuous variables are presented as mean ± standard deviation (for normally distributed data) or median (interquartile range) (for non-normally distributed data), while categorical variables are summarized as frequencies (percentages).To compare differences between groups stratified by NAFLD status, Student’s t-test was used for normally distributed continuous variables with homogeneous variances, the Wilcoxon rank-sum test for non-normally distributed variables, and the chi-square test for categorical variables.

Missing covariate data were handled using multiple imputation by chained equations (MICE).Prior to multivariable modeling, rigorous collinearity screening was performed by calculating the variance inflation factor (VIF>5) for each candidate covariate to assess potential multicollinearity and to optimize model goodness-of-fit and stability. To precisely quantify the independent association between AIP and the risk of prevalent NAFLD, multivariable binary logistic regression models were constructed, with results presented as odds ratios (ORs) and their corresponding 95% confidence intervals (CIs). Considering different levels of confounding, three sequential adjusted models were constructed. Model I adjusted for sex, age, and area of residence. Model II additionally adjusted for marital status, smoking history, history of hypertension, history of diabetes, BMI, and WC. Model III further incorporated laboratory biochemical markers retained after collinearity screening, including WBC count, HGB concentration, ALT, ALP, ALB, UA, FBG,and HbA1c.Rationale for excluding LDL-C from the final model: Although LDL-cholesterol (LDL-C) is a conventional lipid parameter, it is mathematically derived from the Friedewald equation (LDL-C = TC – HDL-C – TG/5), which shares its components (TG and HDL-C) with AIP = log_10_(TG/HDL-C). Including LDL-C in the same model as AIP could therefore introduce overadjustment or collider bias. In pre-analysis VIF screening, LDL-C did not exceed the collinearity threshold (VIF< 5); however, to adhere to strict biological and statistical principles, we performed a sensitivity analysis by re-fitting Model III after excluding LDL-C while retaining all other covariates. Beyond analyzing AIP as a continuous variable to assess the risk associated with each unit increment, AIP was also categorized into quartiles. Using the lowest quartile (Q1) as the reference, adjusted ORs were calculated for each exposure level. Trend tests were performed by entering the median value of each quartile category as a continuous term in the model to verify the existence of a dose-response relationship.

To evaluate effect modification and identify potentially high-risk susceptible populations, prespecified subgroup analyses and interaction tests were conducted. Stratification factors included sex, age group, area of residence, smoking history, history of hypertension, and history of diabetes. Adjusted logistic regression models were run within each subgroup stratum to calculate effect estimates, and interaction P-values were derived by introducing multiplicative interaction terms (AIP × subgroup variable) into the models to determine whether the heterogeneity of association strength across strata was statistically significant.

To further explore potential complex non-linear associations and threshold effects between AIP and NAFLD, restricted cubic spline (RCS) functions were fitted within multivariable logistic regression models. The significance of the non-linear component was assessed using the likelihood ratio test, and a smoothed fitted curve was plotted to intuitively identify potential saturation effects or inflection points.

To further comprehensively evaluate model discrimination, calibration, and clinical utility, this study employed receiver operating characteristic (ROC) curve analysis to assess model discrimination, with the area under the curve (AUC) calculated and AUC differences between models compared using the DeLong test. Calibration curves were plotted to evaluate the agreement between predicted probabilities and observed probabilities. Decision curve analysis (DCA) was performed to calculate the standardized net benefit across a range of risk thresholds, thereby quantifying the net clinical benefit of the models. To quantify the incremental predictive value of AIP beyond traditional risk factors, a baseline model encompassing the full set of demographic, anthropometric, and laboratory indicators (Model 1) was established, and a new model was constructed by further incorporating AIP(Model 2). We calculated the continuous net reclassification improvement (NRI), categorical NRI, and integrated discrimination improvement (IDI). To evaluate model stability and quantify potential overfitting, internal validation was performed using Harrell’s bootstrap optimism correction (1,000 resamples) and repeated 5-fold cross-validation (10 repeats).

All statistical analyses were performed using R software (version 4.2.1) and Free Statistics software (version 2.1.1). A two-tailed P< 0.05 was considered the threshold for statistical significance.

## Results

### Baseline characteristics

A total of 4,390 participants were enrolled in this study, with a mean age of 72.8 ± 6.2 years; 37.7% were male. The overall prevalence of NAFLD was 42.7%.

Baseline characteristics of participants stratified by AIP quartiles are presented in **Table 1**. With increasing AIP levels, NAFLD prevalence exhibited a significant upward trend across quartiles: 22.4%, 36.9%, 50.1%, and 61.3%, respectively (P<0.001). Concurrently, participants with higher AIP levels exhibited a more adverse metabolic profile, including a higher proportion of hypertension and diabetes history, elevated BMI and WC, and increased levels of WBC count, HGB, ALT, UA, FBG, TG, LDL-C, and HbA1c, while HDL-C levels progressively decreased (all P<0.05). Additionally, the age distribution differed across AIP quartile groups, with participants in higher AIP quartiles being relatively younger (P<0.001).

**Table 1:** Baseline characteristics of participants stratified by AIP in relation to NAFLD.

Variables	Total (n=4390)	Q1 (≤-0.562–0.188) (n=1098)	Q2 (0.188–0.381) (n=1097)	Q3 (0.381–0.596) (n=1097)	Q4 (0.596–2.381) (n=1098)	*P*
Male, n (%)	1653 (37.7)	379 (34.5)	420 (38.3)	429 (39.1)	425 (38.7)	0.098
Age(year)	72.8 ± 6.2	73.6 ± 6.6	72.9 ± 6.4	72.6 ± 6.1	72.0 ± 5.6	< 0.001
Married, n (%)	3123 (97.9)	799 (97.8)	775 (98.2)	757 (97.8)	792 (97.9)	0.484
Resident, n (%)						< 0.001
Urban	4068 (92.9)	986 (90)	1021 (93.2)	1033 (94.5)	1028 (93.6)	
Rural	313 (7.1)	109 (10)	74 (6.8)	60 (5.5)	70 (6.4)	
Smoking, n (%)	165 (3.8)	36 (3.3)	40 (3.6)	51 (4.6)	38 (3.5)	0.332
Hypertension history, n (%)	2000 (45.7)	463 (42.4)	476 (43.5)	512 (46.8)	549 (50.2)	0.004
Diabetes history, n (%)	1016 (23.3)	209 (19.2)	242 (22.2)	272 (24.9)	293 (26.8)	< 0.001
WC(cm)	85.3 ± 9.9	82.0 ± 10.5	85.6 ± 10.1	86.7 ± 9.5	86.8 ± 8.8	< 0.001
BMI(kg/m2)	24.2 ± 5.7	23.0 ± 4.0	24.3 ± 6.3	24.7 ± 4.3	24.7 ± 7.4	< 0.001
WBC (10^9^/L)	6.7 ± 2.0	6.2 ± 1.5	6.8 ± 2.8	6.9 ± 1.8	7.0 ± 1.7	< 0.001
HGB (g/L)	133.8 ± 14.1	131.4 ± 14.6	133.1 ± 13.9	135.2 ± 13.9	135.5 ± 13.6	< 0.001
ALT (U/L)	22.3 ± 18.8	20.8 ± 13.1	21.5 ± 11.3	23.0 ± 30.3	23.8 ± 13.9	< 0.001
ALP (U/L)	94.1 ± 51.7	94.4 ± 63.7	94.2 ± 31.7	92.5 ± 31.7	95.4 ± 67.8	0.62
ALB(g/L)	44.0 ± 7.5	43.7 ± 13.8	44.2 ± 3.5	43.8 ± 3.5	44.1 ± 3.0	0.381
HbA1c (%)	6.0 ± 2.8	5.9 ± 2.8	5.8 ± 2.1	6.2 ± 3.3	6.3 ± 2.7	< 0.001
UA (μmol/L)	395.7 ± 162.1	368.2 ± 162.4	382.7 ± 164.2	401.7 ± 104.2	430.1 ± 197.0	< 0.001
FBG (mmol/L)	6.3 ± 2.1	6.0 ± 1.8	6.1 ± 1.7	6.4 ± 2.5	6.7 ± 2.4	< 0.001
LDL-C (mmol/L)	3.3 ± 1.2	3.2 ± 1.3	3.3 ± 0.9	3.4 ± 1.5	3.4 ± 1.0	< 0.001
NAFLD	1874 (42.7)	246 (22.4)	405 (36.9)	550 (50.1)	673 (61.3)	< 0.001

Waist Circumference; BMI, Body Mass Index; WC, Waist Circumference;WBC, White blood cell count; HGB, Hemoglobin concentration; AST, Aspartate Aminotransferase; ALT, Alanine Aminotransferase; ALP, Alkaline Phosphatase; ALB, Albumin; CRP, C-Reactive Protein; HbA1c, Glycated Hemoglobin; UA, Uric Acid; FBG, Fasting Blood Glucose; TG, Triglyceride; TC, Total Cholesterol; HDL-C, High-Density Lipoprotein Cholesterol; LDL-C, Low-Density Lipoprotein Cholesterol.

When stratified by NAFLD status, participants in the NAFLD group were younger, had a lower proportion of males, and were more likely to have a history of hypertension and diabetes compared with the non-NAFLD group (all P<0.001). Moreover, the NAFLD group exhibited higher BMI and WC, accompanied by more pronounced disturbances in glucose and lipid metabolism, as reflected by elevated levels of WBC count, HGB, ALT, ALB, UA, FBG, TG, TC, LDL-C, CRP, HbA1c, and AIP, as well as reduced ALP and HDL-C levels (all P<0.05). No statistically significant differences were observed between the two groups with respect to marital status, smoking history, SBP, DBP,or AST (all P>0.05)(**Supplementary Table 1**).

### Correlation between AIP and NAFLD

Univariate analysis showed that age, gender, marital status, residential location, smoking history, history of hypertension, history of diabetes, BMI, waist circumference, white blood cell count, hemoglobin, ALT, ALB, ALP, uric acid, fasting blood glucose, LDL-C, and HbA1c were associated with NAFLD (P<0.1) (**Supplementary Table 2**). Covariate screening indicated no multicollinearity among these variables (**Supplementary Table 3**).

When AIP was analyzed as a continuous variable, multivariate adjusted analysis (Model III) revealed that each one-unit increment in AIP was significantly associated with an increased risk of NAFLD (adjusted OR = 6.13, 95% CI: 4.55~8.28, P<0.001). After AIP was stratified by quartiles, participants in the highest quartile (Q4) had a significantly higher risk of NAFLD compared with those in the lowest quartile (Q1) (OR = 4.13, 95% CI: 3.2~5.32, P<0.001), with a significant dose–response relationship (P for trend < 0.001) (**Table 2**).A sensitivity analysis additionally including LDL-C confirmed that the AIP odds ratio remained essentially unchanged (OR 6.13, 95% CI 4.55–8.27), with no significant improvement in model fit (likelihood ratio test P = 0.787), supporting the robustness of the primary specification.

**Table 2:** Association between AIP and NAFLD.

NAFLD	Total	Incident rate(%)	Model I	*P*	Model II	*P*	Model III	*P*
AIP	4390	42.7	8.81 (7.02~11.05)	<0.001	7.63 (5.73~10.16)	<0.001	6.13 (4.55–8.28)	<0.001
Quartiles								
Q1	1098	22.4	1 (Ref)		1 (Ref)		1 (Ref)	
Q2	1097	36.9	2.02 (1.67~2.44)	<0.001	1.68 (1.32~2.15)	<0.001	1.69 (1.31~2.18)	<0.001
Q3	1097	50.1	3.46 (2.87~4.17)	<0.001	2.82 (2.22~3.6)	<0.001	2.55 (1.98~3.28)	<0.001
Q4	1098	61.3	5.4 (4.47~6.53)	<0.001	4.82 (3.79~6.13)	<0.001	4.13 (3.2~5.32)	<0.001
P for Trend	1874	42.7	1.74 (1.64~1.84)	<0.001	1.62 (1.52~1.73)	<0.001	1.59 (1.47~1.72)	<0.001

Model I was adjusted for sex, age, and urban/rural residence.

Model II was additionally adjusted for marital status, smoking history, history of hypertension, and history of diabetes.

Model III was further adjusted for all non-collinear covariates. AIP, atherogenic index of plasma; NAFLD, non-alcoholic fatty liver disease; OR, odds ratio; 95% CI, 95% confidence interval; Ref, reference group.

The present study demonstrated that elevated AIP levels were significantly correlated with an increased risk of NAFLD, accompanied by a notable dose–response relationship. AIP was identified as an independent risk factor for NAFLD, and this association remained robust after full adjustment for multiple confounding factors. The findings suggest that AIP has potential clinical value in the risk assessment of NAFLD, which warrants further mechanistic research and cohort studies for validation.

### Subgroup analysis

**Figure 2:**
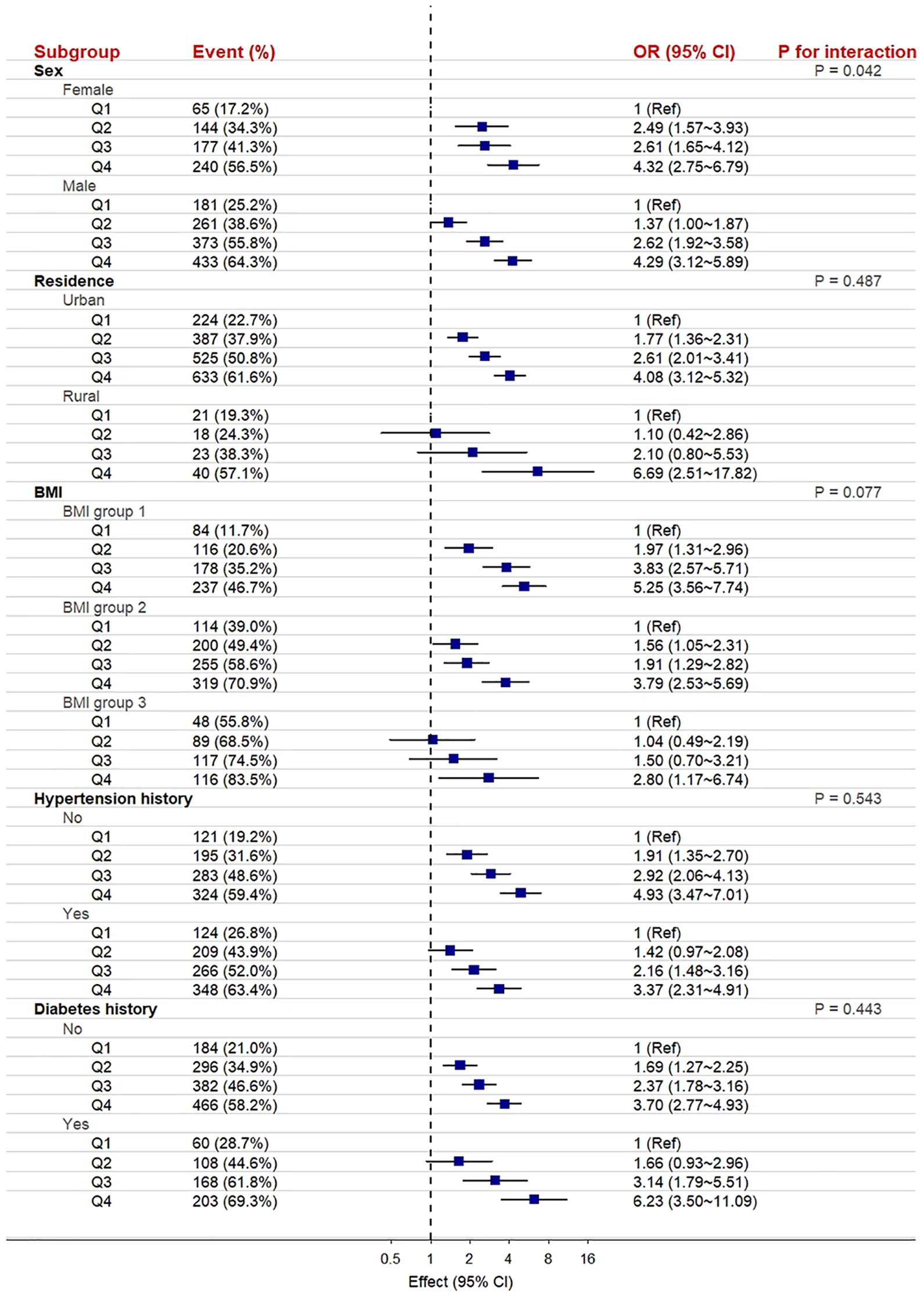
Subgroup analysis of the association between AIP and NAFLD.

As shown in **Figure 2**, a statistically significant interaction by sex was observed (P for interaction = 0.042), while no significant interactions were found across subgroups defined by age group, area of residence, history of hypertension, or history of diabetes (all P for interaction > 0.05), indicating that the association was modified by sex but not by the other factors.

### Threshold effect analysis

**Figure 3:**
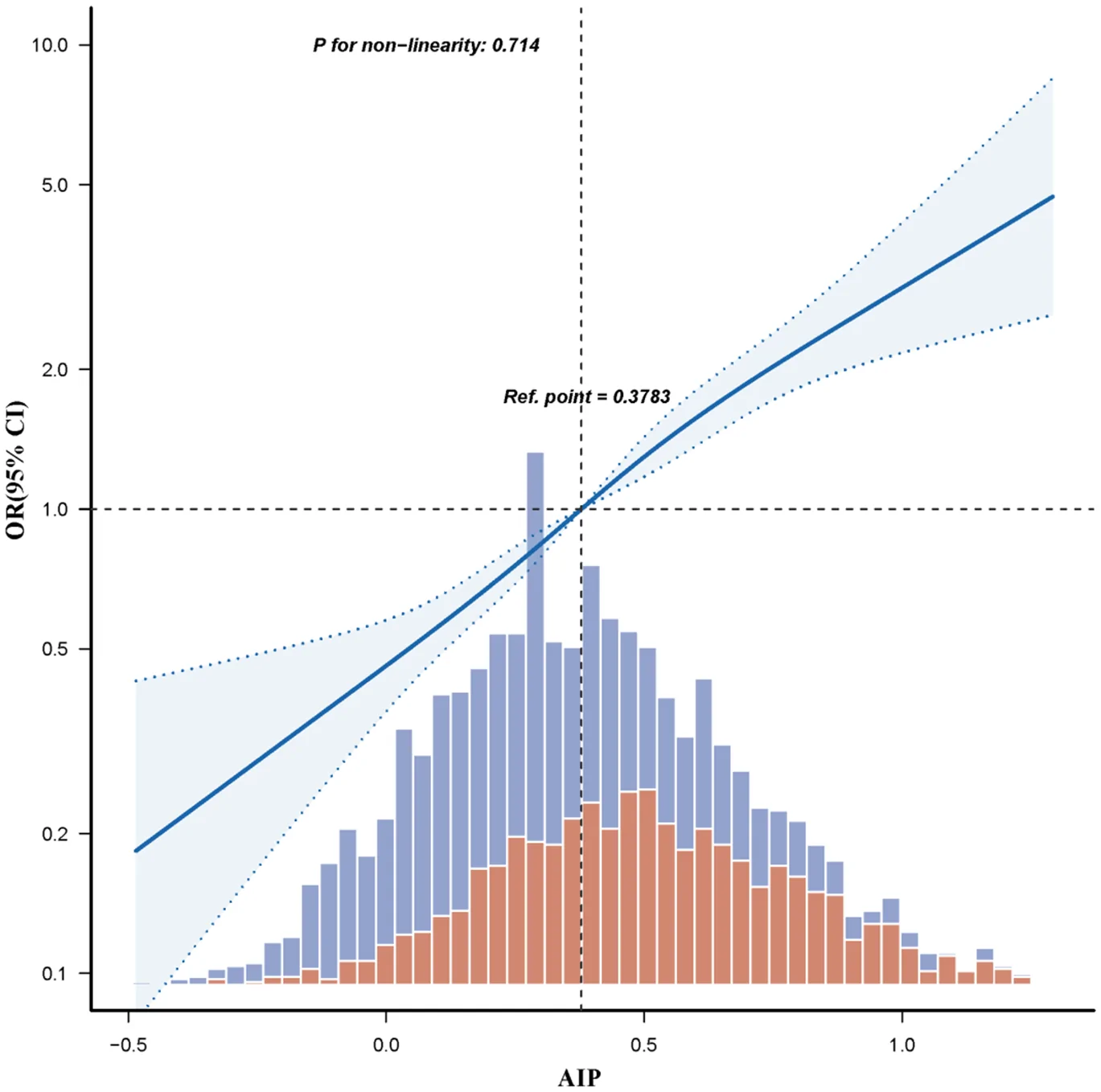
Restricted cubic spline analysis of the association between the AIP and the risk of NAFLD. The solid red line represents the fitted risk curve, and the shaded area indicates the 95% confidence interval. The vertical dashed line denotes the reference point (Ref point = 0.3783, OR = 1.00). The histogram below displays the distribution of AIP values among the study participants. The model was adjusted for age, sex, residence, marital status, smoking history, history of hypertension, history of diabetes, and other non-collinear confounders. The blue stripes represent the frequency distribution of AIP values in the entire study population, while the red stripes represent the frequency distribution of AIP values in a subgroup of patients diagnosed with NAFLD.

Restricted cubic spline (RCS) analysis was applied to examine the dose-response relationship and potential threshold effect between AIP and NAFLD risk. The results demonstrated a linear positive association between AIP and NAFLD risk. With the reference point set at AIP = 0.3783 (OR = 1.00), the risk of NAFLD rose steadily and continuously with increasing AIP levels, without any discernible plateau phase or inflection point. The test for non-linearity yielded P = 0.714, indicating no significant nonlinear association or threshold effect between AIP and NAFLD. The histogram at the bottom of **Figure 3** shows that the AIP values of the study participants were mainly concentrated in the range of −0.5 to 1.0, indicating good population representativeness of the results.

### Predictive performance of AIP for NAFLD

**Figure 4:**
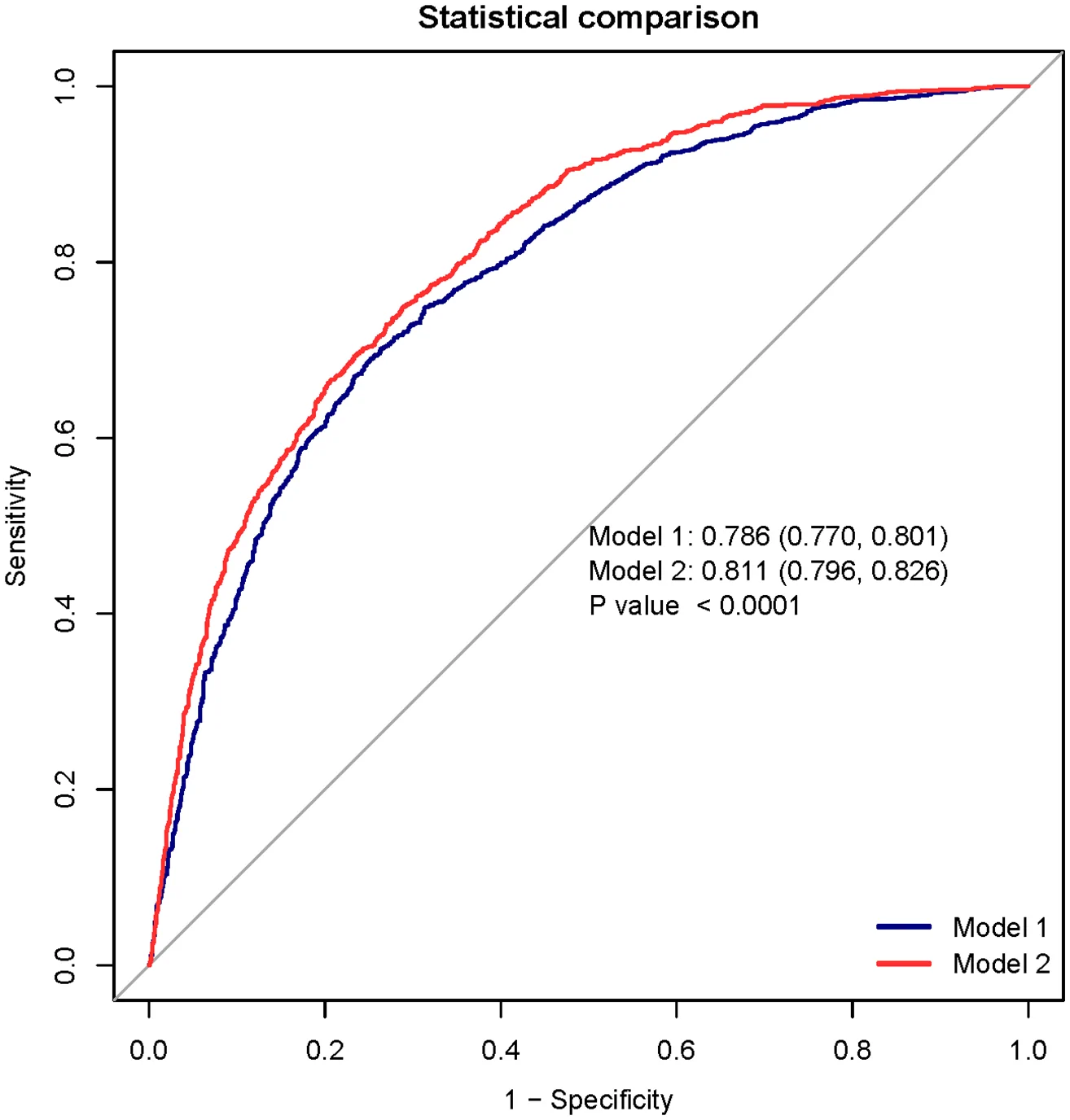
ROC curves of the NAFLD prediction models. Model 1 (base model) incorporated 17 traditional NAFLD risk factors, including age, sex, residence, history of hypertension, history of diabetes, BMI, waist circumference, and a comprehensive panel of laboratory biochemical indicators without LDL-C. Model 2 (new model) additionally included AIP.

**Figure 5:**
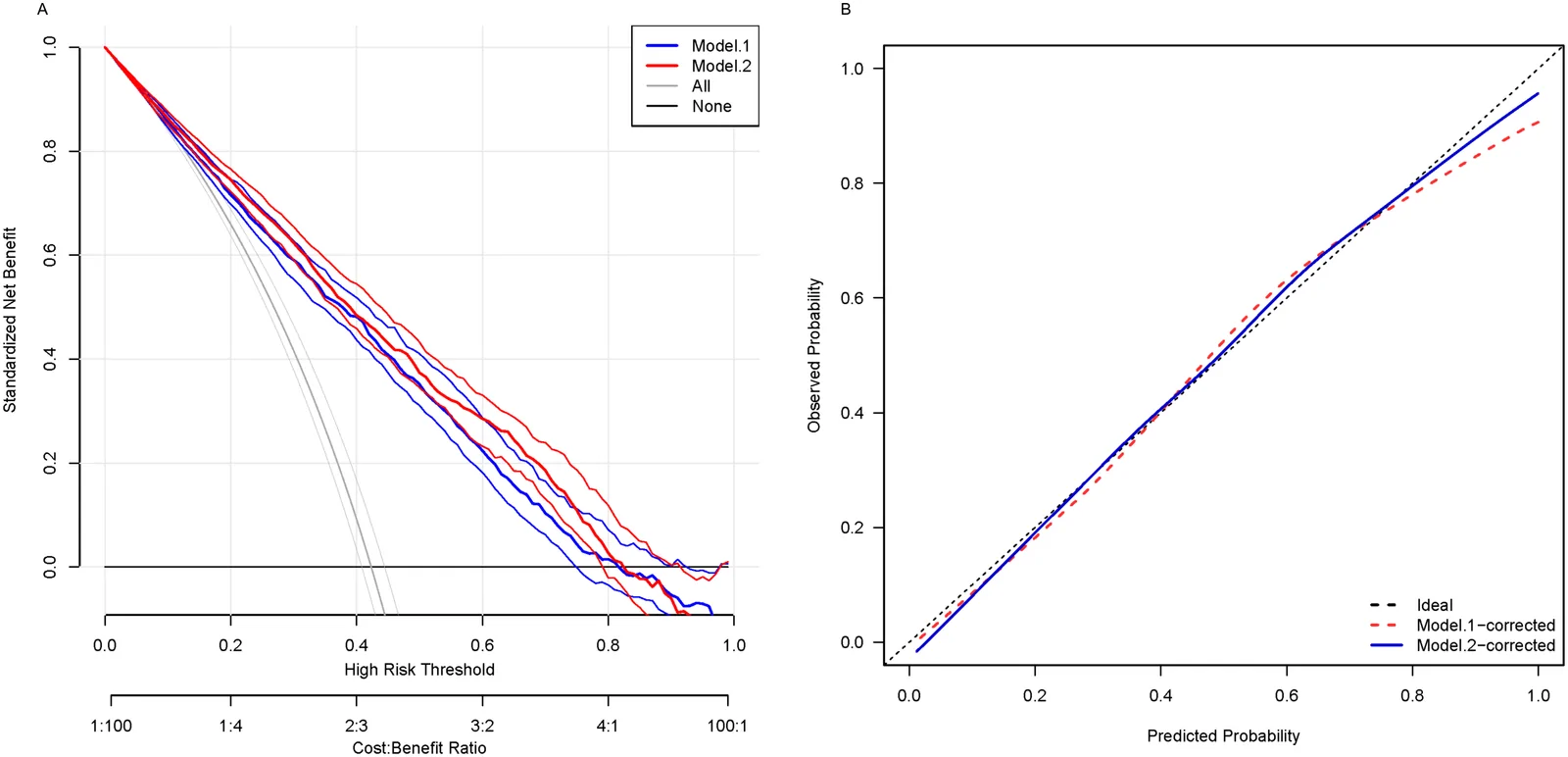
Decision curve analysis **(A)** and calibration curves **(B)** of the NAFLD prediction models. **(A)** Comparison of the clinical utility of the two NAFLD risk prediction models. The y-axis represents the standardized net benefit; the lower x-axis represents the threshold probability, and the upper x-axis represents the corresponding cost-benefit ratio. The gray lines denote the two extreme strategies of “intervention for none” and “intervention for all.” Within the clinically reasonable threshold probability range (0.05–0.95), the standardized net benefit of Model 2 was consistently higher than that of Model 1. **(B)** The dashed line (“Ideal”) represents perfect calibration, and the solid lines represent the corrected calibration curves of the models. Both models demonstrated good calibration; Model 2 was closer to the ideal line across the full probability range, particularly in the intermediate-to-high risk interval of 0.4–0.8.

To further quantify the predictive value of the atherogenic index of plasma (AIP) for non-alcoholic fatty liver disease (NAFLD) in community-dwelling older adults, this study systematically evaluated the base model (Model 1) and the new model (Model 2) using receiver operating characteristic (ROC) curve analysis (**Figure 4**), calibration curve analysis (**Figure 5A**), and decision curve analysis (**Figure 5B**) to comprehensively assess model discrimination, calibration, and clinical utility.

ROC curve analysis demonstrated that both models exhibited favorable predictive performance for NAFLD in the community-dwelling elderly population (**Figure 4**). The area under the curve (AUC) for the base model (Model 1) was 0.786 (95% CI: 0.770–0.801), indicating that the combination of traditional risk factors could already reasonably discriminate between NAFLD and non-NAFLD individuals. After incorporating AIP, the AUC of Model 2 was significantly increased to 0.811 (95% CI: 0.796–0.826), with the difference reaching high statistical significance (P< 0.001). Further analysis revealed that the addition of AIP improved overall model discrimination by 3.2%, an incremental gain that is particularly noteworthy in the elderly population. Compared with individual lipid parameters, AIP, as a composite marker integrating triglycerides (TG) and high-density lipoprotein cholesterol (HDL-C), can more comprehensively capture the overall state of lipid metabolic dysregulation, thereby providing supplementary information for NAFLD risk prediction.

Calibration curves were used to assess the agreement between predicted probabilities and observed probabilities, reflecting the consistency between predicted and actual event rates. As shown in **Figure 5**, both model curves closely followed the ideal diagonal line, indicating that both models possessed good calibration and that no significant deviation existed between predicted and observed probabilities. Further comparison showed that the calibration curve of the optimized model (Model 2-corrected) adhered more closely to the ideal diagonal line across the full probability range, particularly in the intermediate-to-high risk interval (predicted probability 0.4–0.8), where its prediction bias was notably smaller than that of the base model. This suggests that the inclusion of AIP not only improves the model’s ability to discriminate among individuals at different risk levels but also more accurately quantifies their actual disease risk, thereby circumventing the overestimation or underestimation that tends to occur with traditional models in intermediate-to-high risk populations, and providing a more precise reference for individualized clinical risk assessment.

Discrimination and calibration only reflect the statistical performance of a model, whereas decision curve analysis (DCA) can directly quantify clinical utility by calculating the standardized net benefit across a range of risk thresholds. As shown in **Figure 5**, over the vast majority of clinically reasonable threshold probabilities (0.05–0.95), the standardized net benefit of both models was substantially higher than that of the two extreme strategies—”intervention for all” and “intervention for none”—confirming the fundamental clinical value of NAFLD risk assessment based on traditional risk factors. More importantly, across the entire threshold range, the optimized model incorporating AIP consistently exhibited higher standardized net benefit than the base model. This implies that, at a given risk threshold, employing the optimized model for NAFLD screening and risk stratification can identify a greater number of truly high-risk individuals without increasing the false-positive rate. Internal validation confirmed robust model stability: Harrell’s bootstrap optimism correction (1,000 resamples) yielded an optimism-corrected AUC of 0.808 (apparent AUC 0.811; optimism 0.003), and repeated 5-fold cross-validation (10 repeats) produced a mean AUC of 0.804 (SD = 0.015).

### Incremental analysis

To assess whether AIP offers independent incremental predictive value beyond traditional risk factors, we computed NRI and IDI by adding AIP to Model 1 (with all covariates). As shown in **Table 3**, incorporating AIP significantly improved prediction: categorical NRI = 0.0997 [0.0668-0.1325], continuous NRI = 0.4623 [0.3931-0.5314], IDI = 0.0487 [0.0409-0.0565], all P< 0.001. AIP provides unique information on atherogenic lipid dysregulation and independently enhances individualized NAFLD risk prediction in the elderly.

**Table 3:** Incremental predictive value of AIP for NAFLD beyond traditional risk factors.

Metric	Estimate (95% CI)	*P value*
Categorical NRI	0.0997 [0.0668-0.1325]	<0.001
Continuous NRI	0.4623 [0.3931-0.5314]	<0.001
IDI	0.0487 [0.0409-0.0565]	<0.001

The baseline model (Model 1) included all traditional covariates: age, sex, residence, marital status, smoking, hypertension history, diabetes history, BMI, WC, WBC, HGB, ALT, ALP, ALB, UA, FBG, and HbA1c. The new model (Model 2) added AIP to Model 1.

NRI, net reclassification improvement; IDI, integrated discrimination improvement; CI, confidence interval.

All P values and 95% CIs were derived from 1,000 bootstrap resamples.

### Comparison of AIP with other NAFLD biomarkers

**Figure 6:**
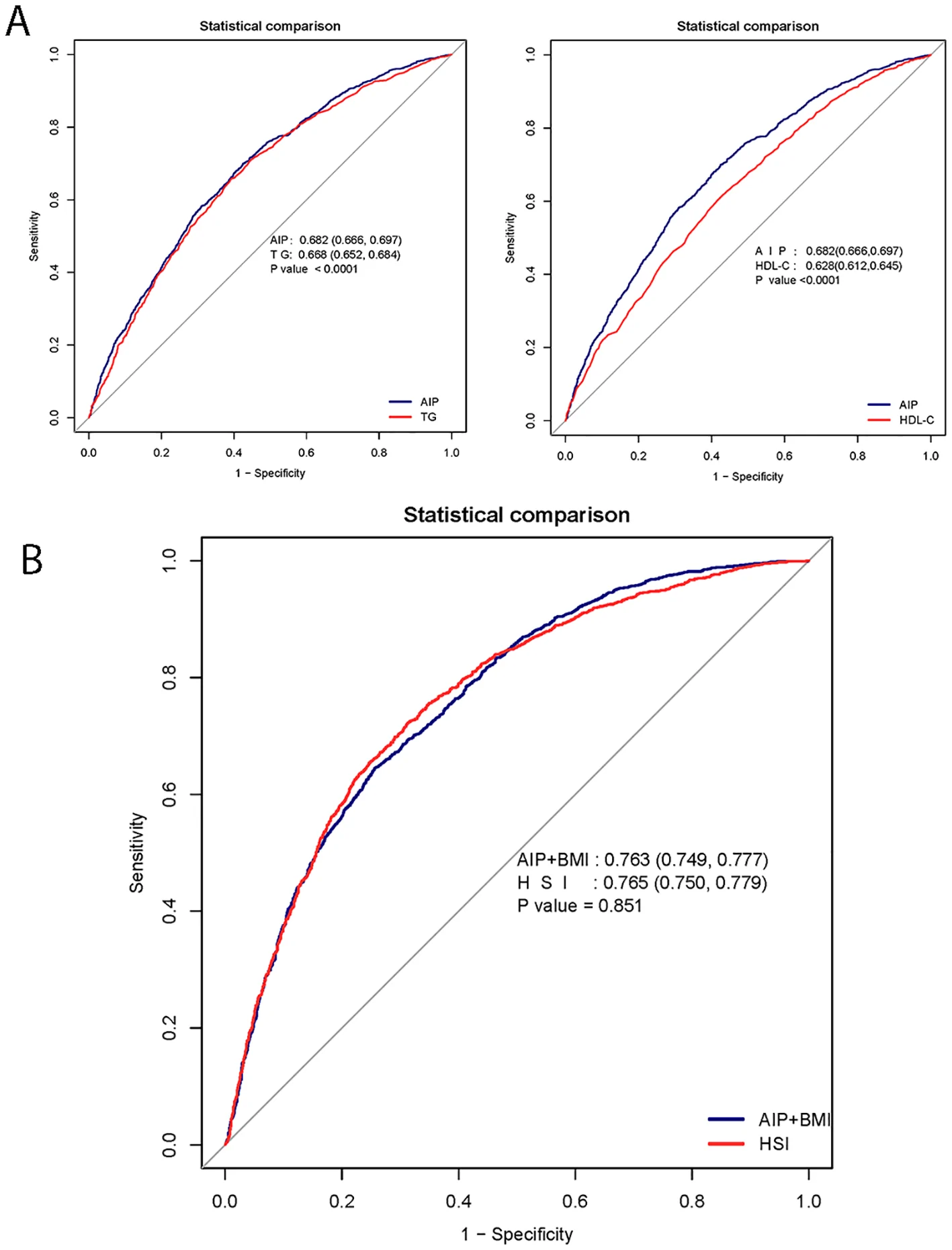
Comparison of receiver operating characteristic (ROC) curves for NAFLD prediction. **(A)** AIP versus its individual components TG and HDL-C. AIP demonstrated significantly better discriminatory performance than either TG or HDL-C alone. **(B)** AIP+BMI versus the hepatic steatosis index (HSI). The AIP+BMI composite model showed comparable AUC to HSI, a five-variable scoring system.

To further contextualize the predictive utility of AIP, we compared its discriminatory performance against its individual lipid components (TG and HDL-C) and against the validated hepatic steatosis index (HSI). As shown in **Figure 6A**, AIP demonstrated superior AUC compared with either TG or HDL-C alone for NAFLD prediction (AIP: 0.682 [95% CI: 0.666-0.697] vs. TG: 0.668 [95% CI: 0.652-0.684] vs. HDL-C: 0.628 [95% CI: 0.612-0.645]; both DeLong P< 0.0001), confirming that the composite index captures synergistic information beyond its individual components. When compared with HSI—a multi-variable scoring system incorporating ALT, AST, BMI, sex, and diabetes status—AIP alone showed lower AUC than HSI (0.682 vs. 0.765; P< 0.001). However, after adding BMI to AIP (AIP+BMI), the combined model achieved an AUC comparable to HSI (0.763 vs. 0.765; DeLong P = 0.851), indicating that a two-variable model (AIP+BMI) can approximate the discriminatory performance of a five-variable scoring system (**Figure 6B**). These findings suggest that AIP, particularly when combined with BMI, offers a practical and parsimonious alternative for NAFLD risk stratification in community-based elderly screening. When compared with the TyG index, a widely used surrogate of insulin resistance, AIP showed comparable discriminatory performance (AUC 0.682 vs. 0.687; DeLong P = 0.28; **Supplementary Figure 1**).

## Discussion

This large-scale, community-based cross-sectional study of 4,390 Chinese older adults revealed several important findings. The prevalence of NAFLD was 42.7%. This prevalence increased progressively across AIP quartiles. After comprehensive adjustment for demographic, anthropometric, lifestyle, and laboratory confounders, AIP remained independently associated with NAFLD risk. Each 1-unit increment in AIP was associated with a 6.13-fold higher risk. Participants in the highest AIP quartile had a 4.13-fold increased risk compared with those in the lowest quartile. A significant dose-response trend was observed. Restricted cubic spline analysis confirmed a strictly linear, threshold-free positive association. Subgroup analyses demonstrated consistent findings across age, residence, hypertension, and diabetes strata, with a statistically significant interaction by sex (P = 0.042). Adding AIP to a baseline model already containing 17 traditional risk factors significantly improved model performance. Discrimination improved from an AUC of 0.786 to 0.811. Calibration remained excellent. Decision curve analysis showed greater clinical net benefit. Risk reclassification also improved significantly. The categorical NRI was 0.0997 and the IDI was 0.0487. These results establish AIP as an independent, linearly associated risk marker for NAFLD in the elderly. They also support its use for integrated cardiometabolic risk stratification.

Our findings are broadly consistent with earlier reports in general adult populations. Li et al. reported an independent, dose-dependent positive association between AIP and hepatic steatosis in a nationally representative US sample (28).Liu et al. showed that AIP outperforms individual lipid parameters for NAFLD prediction (9).Chen et al. observed a similar association in a predominantly young and middle-aged cohort (18).However, none of these studies specifically focused on the elderly. They also did not systematically characterize the shape of the dose-response relationship. Similarly, Chen et al. observed an elevated AIP associated with MAFLD prevalence in US adults using NHANES 2017–2018 data (18), and Shen et al. found AIP independently associated with MAFLD in hypertensive patients (OR = 3.48, 95% CI: 2.39–5.06) (16). However, most prior studies were conducted in general adult populations, and whether these associations hold specifically in older adults has remained unclear. Pan et al. developed a NAFLD risk prediction model for older Chinese adults but did not specifically evaluate AIP (29), and Samimi et al. identified AIP as an independent predictor of metabolic-associated fatty liver disease without exploring the effect of aging (14). Our study directly addresses these gaps by demonstrating that the AIP-NAFLD association is not only preserved but appears notably robust in the elderly, with a strong continuous effect size (OR = 6.13 per 1-unit increment) and a strictly linear, threshold-free relationship across the full AIP spectrum. This suggests that, unlike some biomarkers that exhibit ceiling effects or J-shaped associations in older populations, the risk conferred by increasing AIP is consistent across the entire range in elderly individuals.

A key finding of our study is that AIP offers significant incremental predictive value beyond conventional risk factors for NAFLD risk stratification in older adults. Beyond the improvement in AUC (from 0.786 to 0.811) and the significant reclassification indices, we conducted comprehensive comparative ROC analyses. AIP (AUC = 0.682) demonstrated superior discriminatory performance compared with its individual constituent lipid parameters, TG (AUC = 0.668) and HDL-C (AUC = 0.628; both P< 0.001). AIP and the TyG index showed comparable AUCs (0.682 vs. 0.687; DeLong P > 0.05), confirming equivalent diagnostic performance. The hepatic steatosis index (HSI) alone showed a higher AUC (0.765 vs. 0.682 for AIP alone; P< 0.001); however, when BMI was added to AIP, the combined model achieved an AUC of 0.763, which was statistically comparable to HSI (0.765; P = 0.851), suggesting that AIP combined with BMI offers a practical, low-cost alternative to multi-variable screening tools. These findings extend previous reports: Liu et al. showed that AIP outperformed TG, TC, LDL-C, HDL-C, and non-HDL-C in identifying NAFLD (9), while Huang et al. reported that AIP had the highest AUC for carotid atherosclerosis among lipid parameters (15). Collectively, our data suggest that AIP retains robust discriminative ability even in the elderly, where age-related metabolic changes may attenuate the performance of certain biomarkers.

The biological mechanisms linking AIP to NAFLD are multifaceted. AIP serves as a reliable surrogate for the proportion of small dense LDL (sdLDL) particles, which are more atherogenic and more readily taken up by hepatocytes via scavenger receptors, promoting hepatic lipid deposition (30, 31). Elevated sdLDL also reflects enhanced cholesteryl ester transfer protein activity and impaired reverse cholesterol transport, further exacerbating intrahepatic lipid accumulation. Beyond its direct reflection of atherogenic lipoprotein abnormalities, AIP is strongly correlated with systemic insulin resistance (12). In the insulin-resistant state, adipose tissue lipolysis is inadequately suppressed, increasing the flux of free fatty acids to the liver, where they drive *de novo* lipogenesis and impair mitochondrial fatty acid β-oxidation, creating a vicious cycle of hepatic lipid accumulation (32). In older adults, age-related declines in insulin sensitivity, progressive visceral adiposity, and diminished lipid oxidative capacity may further accentuate this relationship. The strictly linear dose-response relationship observed in our study supports a continuous biological gradient rather than a threshold phenomenon, consistent with the interpretation that even modest AIP elevations contribute incrementally to NAFLD risk in this vulnerable population.

Our findings have practical implications for NAFLD screening and cardiometabolic risk management in aging populations. AIP can be calculated from routine lipid profiles at no additional cost, making it highly scalable in primary care and community health settings. Given that NAFLD shares pathogenic pathways with cardiovascular disease (6), AIP may serve as a unifying risk marker that simultaneously informs hepatic and cardiovascular risk assessment. Indeed, AIP is an established predictor of atherosclerotic cardiovascular disease (10) and stroke (11), and recent evidence also links AIP to kidney failure risk (22). Clinicians should interpret AIP as a continuous risk indicator rather than applying a binary threshold (29). For example, individuals in the highest AIP quartile had approximately a 4-fold increased NAFLD risk and may reasonably be prioritized for abdominal ultrasonography and comprehensive metabolic evaluation. Moreover, given that AIP reflects the link between lipid metabolism and insulin resistance, elevated AIP may flag older adults who could benefit from early lifestyle modifications, including dietary changes and increased physical activity, prior to the onset of overt metabolic disease.

This study has several notable strengths. The large sample size (n = 4,390) from a routine community health examination cohort provides good representativeness of community-dwelling older adults in western Guangdong. We performed rigorous adjustment for extensive covariates, formal multicollinearity screening, restricted cubic spline analysis, and a comprehensive model evaluation framework encompassing ROC analysis, calibration plots, decision curve analysis, and reclassification statistics. However, several limitations should be acknowledged. The cross-sectional design precludes causal inference, and reverse causation cannot be excluded. The predictive model was developed and internally validated in a single community-based cohort. External validation in geographically and demographically diverse populations is warranted prior to clinical implementation. NAFLD was diagnosed by abdominal ultrasonography rather than MRI or liver biopsy, which may have limited sensitivity for mild steatosis. The study population was restricted to a single geographic region in southern China, limiting generalizability to other ethnic groups or younger populations. Data on lipid-lowering and antidiabetic medications were unavailable, although this limitation reflects real-world community conditions. Residual confounding by unmeasured variables remains possible. Prospective cohort studies are needed to confirm the temporal relationship between AIP and incident NAFLD in the elderly, and future research should evaluate whether AIP-guided screening strategies improve clinical outcomes. In conclusion, AIP demonstrates an independent, strictly linear association with NAFLD risk in community-dwelling older adults, exhibiting no discernible safe threshold. This simple, low-cost biomarker may serve as a useful tool for NAFLD risk stratification in aging populations.

## Data Availability

The raw data supporting the conclusions of this article will be made available by the authors, without undue reservation.
