# Supplementary material for: Atherogenic index of plasma as a simple marker for NAFLD risk stratification in community-dwelling older adults: a cross-sectional study with incremental predictive value assessment
Source: Front Endocrinol (Lausanne). 2026 Aug 26;17:1907673. doi: 10.3389/fendo.2026.1907673 (PMC13553247; doi:10.3389/fendo.2026.1907673)
Supplement: Supplementary file 1 [file Table1.docx]

# **Supplementary** Table 1 Baseline Characteristics of Participants Stratified by NAFLD status.

| **Variables** | **Total (n=4390 )** | **Non-NAFLD (n= 2516)** | **NAFLD**  **(n=1874)** | ***P*** |
| --- | --- | --- | --- | --- |
| **Male, n (%)** | 1653 (37.7) | 1027 (40.8) | 626 (33.4) | < 0.001 |
| **Age(year)** | 72.8 ± 6.2 | 73.5 ± 6.6 | 71.9 ± 5.6 | < 0.001 |
| **Married, n (%)** | 3123 (71.1) | 1787 (71) | 1336 (71.3) | <0.578 |
| **Resident, n (%)** |  |  |  | < 0.001 |
| **Urban** | 4068（92.9） | 2299（91.6） | 1769（94.5） |  |
| **Rural** | 313（7.1） | 211（8.4） | 102（5.5） |  |
| **Smoking, n (%)** | 165 ( 3.8) | 99 (3.9) | 66 (3.5) | <0.477 |
| **Hypertension history, n (%)** | 2000 (45.6) | 1053 (41.9) | 947 (50.5) | < 0.001 |
| **Diabetes history, n (%)** | 1016 (23.1) | 477 (19) | 539 (28.8) | < 0.001 |
| **WC(cm)** | 85.3 ± 9.9 | 82.5 ± 9.5 | 88.9 ± 9.2 | < 0.001 |
| **BMI(kg/m^2^)** | 24.2 ± 5.7 | 23.1 ± 5.0 | 25.7 ± 6.2 | < 0.001 |
| **WBC (10^9^/L)** | 6.7 ± 2.0 | 6.6 ± 2.3 | 6.9 ± 1.6 | < 0.001 |
| **HGB (g/L)** | 133.8 ± 14.1 | 132.1 ± 14.1 | 136.2 ± 13.7 | < 0.001 |
| **ALT (U/L)** | 22.3 ± 18.8 | 20.4 ± 21.7 | 24.7 ± 13.7 | < 0.001 |
| **ALP (U/L）** | 94.1 ± 51.7 | 95.8 ± 62.5 | 91.9 ± 32.1 | <0.015 |
| **ALB(g/L)** | 44.0 ± 7.5 | 43.7 ± 9.6 | 44.2 ± 3.1 | <0.031 |
| **CRP (mg/L)** | 3.3 ± 6.5 | 3.1 ± 7.0 | 3.6 ± 5.8 | <0.049 |
| **HbA1c (%)** | 6.0 ± 2.8 | 5.8 ± 2.5 | 6.3 ± 3.1 | < 0.001 |
| **UA (μmol/L)** | 395.7 ± 162.1 | 378.1 ± 144.6 | 419.3 ± 180.3 | < 0.001 |
| **FBG (mmol/L)** | 6.3 ± 2.1 | 6.1 ± 1.8 | 6.7 ± 2.4 | < 0.001 |
| **TG (mmol/L)** | 2.0 ± 3.6 | 1.7 ± 4.5 | 2.3 ± 1.8 | < 0.001 |
| **TC (mmol/L)** | 6.0 ± 7.9 | 5.8 ± 1.4 | 6.3 ± 11.9 | <.041 |
| **HDL-C (mmol/L)** | 1.5 ± 0.4 | 1.6 ± 0.5 | 1.4 ± 0.4 | < 0.001 |
| **LDL-C (mmol/L)** | 3.3 ± 1.2 | 3.3 ± 1.1 | 3.4 ± 1.3 | <0.013 |
| **AIP** | 0.4 ± 0.3 | 0.3 ± 0.3 | 0.5 ± 0.3 | < 0.001 |

Abbreviations: Waist Circumference; BMI: Body Mass Index; WC: Waist Circumference;WBC: White blood cell count; HGB: Hemoglobin concentration; AST: Aspartate Aminotransferase; ALT: Alanine Aminotransferase; ALP: Alkaline Phosphatase; ALB: Albumin; CRP: C-Reactive Protein; HbA1c: Glycated Hemoglobin; UA: Uric Acid; FBG: Fasting Blood Glucose; TG: Triglyceride; TC: Total Cholesterol; HDL-C: High-Density Lipoprotein Cholesterol; LDL-C: Low-Density Lipoprotein Cholesterol.
