# Supplementary material for: Atherogenic index of plasma as a simple marker for NAFLD risk stratification in community-dwelling older adults: a cross-sectional study with incremental predictive value assessment
Source: Front Endocrinol (Lausanne). 2026 Aug 26;17:1907673. doi: 10.3389/fendo.2026.1907673 (PMC13553247; doi:10.3389/fendo.2026.1907673)
Supplement: Supplementary file 2 [file Table2.docx]

**Supplementary table 2.Univariate logistic regression analysis of factors associated with NAFLD.**

| **Variable** | **OR_95CI** | **P** |
| --- | --- | --- |
| **Male, n (%)** | 1.38(1.21~1.56) | <0.001 |
| **Age(year)** | 0.96(0.95~0.97) | <0.001 |
| **Married, n (%)** | 0.76(0.46~1.27) | 0.298 |
| **Rural, n (%)** | 0.63(0.49~0.80) | <0.001 |
| **Smoking, n (%)** | 0.89(0.65~1.22) | 0.477 |
| **Hypertension history, n (%)** | 1.41(1.25~1.60) | <0.001 |
| **Diabetes**  **history, n (%)** | 1.72(1.49~1.98) | <0.001 |
| **WC(cm)** | 1.09(1.08~1.09) | <0.001 |
| **BMI(kg/m2)** | 1.23(1.21~1.26) | <0.001 |
| **WBC (109/L)** | 1.12(1.08~1.16) | <0.001 |
| **HGB (g/L)** | 1.02(1.02~1.03) | <0.001 |
| **AST (U/L)** | 1.00(0.99~1.00) | 0.342 |
| **ALT (U/L)** | 1.03(1.02~1.03) | <0.001 |
| **ALP (U/L）** | 1.00(1.00~1.00) | 0.015 |
| **ALB(g/L)** | 1.02(1.01~1.04) | 0.011 |
| **CRP (mg/L)** | 1.01(1.00~1.02) | 0.052 |
| **HbA1c (%)** | 1.08(1.05~1.11) | <0.001 |
| **UA (μmol/L)** | 1.00(1.00~1.00) | <0.001 |
| **FBG (mmol/L)** | 1.17(1.13~1.20) | <0.001 |
| **TC (mmol/L)** | 1.07(1.03~1.12) | 0.001 |
| **LDL-C (mmol/L)** | 1.07(1.01~1.14) | 0.017 |
