# Supplementary material for: Atherogenic index of plasma as a simple marker for NAFLD risk stratification in community-dwelling older adults: a cross-sectional study with incremental predictive value assessment
Source: Front Endocrinol (Lausanne). 2026 Aug 26;17:1907673. doi: 10.3389/fendo.2026.1907673 (PMC13553247; doi:10.3389/fendo.2026.1907673)
Supplement: Supplementary file 3 [file Table3.docx]

**Supplementary table 3.Variance inflation factor (VIF) values for all candidate predictors.**

| **Variable** | **VIF** |
| --- | --- |
| **Male, n (%)** | 1.553 |
| **Age(year)** | 1.081 |
| **Married, n (%)** | 1.049 |
| **Rural, n (%)** | 1.076 |
| **Smoking, n (%)** | 1.087 |
| **Hypertension history, n (%)** | 1.096 |
| **Diabetes history, n (%)** | 1.519 |
| **WC(cm)** | 1.507 |
| **BMI(kg/m2)** | 1.428 |
| **WBC (109/L)** | 1.093 |
| **HGB (g/L)** | 1.345 |
| **ALT (U/L)** | 1.058 |
| **ALP (U/L）** | 1.101 |
| **ALB(g/L)** | 1.021 |
| **HbA1c (%)** | 1.16 |
| **UA (μmol/L)** | 1.156 |
| **FBG (mmol/L)** | 1.541 |
| **LDL-C (mmol/L)** | 1.051 |
